# TRP Channels as Cellular Targets of Particulate Matter

**DOI:** 10.3390/ijms22052783

**Published:** 2021-03-09

**Authors:** Alina Milici, Karel Talavera

**Affiliations:** Laboratory of Ion Channel Research, Department of Cellular and Molecular Medicine, KU Leuven, VIB Center for Brain & Disease Research, 3000 Leuven, Belgium; milicialina13@gmail.com

**Keywords:** TRP channel, TRPA1, TRPV1, TRPV4, TRPM2, TRPM8, particular matter, nanoparticle, smoke, diesel

## Abstract

Particulate matter (PM) is constituted by particles with sizes in the nanometer to micrometer scales. PM can be generated from natural sources such as sandstorms and wildfires, and from human activities, including combustion of fuels, manufacturing and construction or specially engineered for applications in biotechnology, food industry, cosmetics, electronics, etc. Due to their small size PM can penetrate biological tissues, interact with cellular components and induce noxious effects such as disruptions of the cytoskeleton and membranes and the generation of reactive oxygen species. Here, we provide an overview on the actions of PM on transient receptor potential (TRP) proteins, a superfamily of cation-permeable channels with crucial roles in cell signaling. Their expression in epithelial cells and sensory innervation and their high sensitivity to chemical, thermal and mechanical stimuli makes TRP channels prime targets in the major entry routes of noxious PM, which may result in respiratory, metabolic and cardiovascular disorders. On the other hand, the interactions between TRP channel and engineered nanoparticles may be used for targeted drug delivery. We emphasize in that much further research is required to fully characterize the mechanisms underlying PM-TRP channel interactions and their relevance for PM toxicology and biomedical applications.

## 1. Introduction

Particulate matter (PM) is class of small-sized material with dimensions ranging from the micrometer to nanometer scale. They include fine particles of less than 10 μm and 2.5 μm in diameter (PM_10_ and PM_2.5_) and ultrafine particles (UFP) or nanoparticles (NPs), with less than 100 nm in one dimension. For comparison, the diameter of a human hair is 50–70 μm in diameter. The small size of PM matter allows it to enter our bodies, cross different barriers, deposit in tissues and interact with cellular and subcellular structures. Some micro- and nano-sized particles are not harmful and are specially engineered to be used in medicine as agents for targeting specific cell types, for instance for drug delivery [1,2]. However, in most cases, particulates produce unwanted effects, which are not yet fully understood because of the complexity of their interactions with biological tissue [3,4]. The vast majority of PM are produced as by-products from other processes and are believed, and in most of the cases proved, to have deleterious health effects. For instance, the IARC (International Agency for Research on Cancer) and the WHO (World Health Organization) classified airborne particles as a group 1 carcinogen agent [5]. Sources of PM include natural origins such as dust and fires, pollution caused by the combustion of fossil fuels and numerous industries ranging from pharmaceuticals to cosmetics, foods and construction [6].

The interaction mechanisms and the effects caused by PM in biological tissues depend in a great measure on their physical and chemical properties. Fine and ultrafine particles differ, among other features, in their size, composition, charge, solubility and surface-to-volume ratio. Particles with a smaller diameter can penetrate in deeper biological structures, and a larger surface-to-volume ratio facilitates more interaction with the molecules they come in contact with. The chemical structure of PM offers them very particular properties and allows particles to interact with specific structures, while the surface charge leads to electrostatic forces between PM and electrically charged and polar microscopic structures in our bodies. The vast majority of studies show that the response triggered in cells by NPs is directly proportional to the surface charge and concentration and inversely proportional to their size. The tuning of these can result in better performance of particles engineered for specific biological and medical applications, but for the same reasons may also cause more cellular damage [1,7].

PM can enter our bodies through different routes including the skin, intravenous injections and ingestion, but most of them are inhaled. Once inside the body, they interact with the plasma membrane of cells and the smallest can make their way into cellular organelles [8]. One category of structures they can interact with in the cellular membrane are the Transient Receptor Potential (TRP) proteins. These constitute a superfamily of cation channels that in mammals can be divided into 6 subfamilies based on their amino acid sequence similarities, which however, not always reflects common functional properties, such as activation and modulation mechanisms. These are TRPC (Canonical 1–7), TRPV (Vanilloid 1–6), TRPM (Melastatin 1–8), TRPA (Ankyrin 1), TRPP (Polycystic 1–3), and TRPML (Mucolopin 1–3) [9,10]. The structures of TRP channels consist of homo- or hetero-tetramers of six transmembrane helical domains (TM1 to TM6), with both C- and N-termini situated intracellularly and a pore-loop region situated between the fifth (TM5) and sixth (TM6) transmembrane domains. Activation of TRP channels leads to cation influx, mainly Ca^2+^ and Na^+^, which results in the increase of intracellular concentrations of these ions and membrane depolarization. Thus, they regulate intracellular biochemical signaling processes and cellular electrical excitability [11].

TRP channels can be activated and modulated by a wide variety of stimuli, including chemical agonists, temperature changes, mechanical stress, osmotic pressure and G-protein coupled receptor activation. They play important roles in multiple physiological processes, including nociception [12,13,14], thermosensation [15,16], chemosensation [17], regulation of the vascular tone and permeability [18,19,20], release of neuropeptides [21] and immune cell mediators [22], ciliary beating [23,24,25], mucus secretion [26,27], and barrier function in the airways and the skin [28,29,30,31].

The most researched TRP channel in terms of their interaction with PM are TRPV1 and TRPV4. These channels are activated by heating, oxidative stress, osmotic pressure, mechanical perturbations and fatty acid metabolites [32,33,34,35,36,37]. TRPV1 is notably activated by the pungent natural compound capsaicin and by acidosis [38], whereas TRPV4 is best known for being activated by phorbol derivatives (4α-phorbol 12,13-didecanoate, AKA 4αPDD) and the synthetic agonist GSK1016790A [39]. The mammalian TRPA subfamily has only one member, TRPA1, which owes its name to the presence of 14 ankyrin repeats in the N-terminus. The most salient functional feature of TRPA1 is that it is activated by multiple electrophilic compounds and a plethora of other chemically unrelated amphiphilic compounds. TRPA1 has been reported to be activated by multiple factors, including Ca^2+^, trace metals, pH changes, and reactive oxygen, nitrogen, and carbonyl species [40,41], as well as by endogenous factors released upon tissue damage [42]. Within the TRPM family, PM predominantly interacts with TRPM2 and TRPM8. The main known activators of TRPM2 are ADP-ribose and the oxidative stress [33,43,44,45]. TRPM8 is sensitive to cold temperatures and compounds that produce cooling sensations, such as menthol, eucalyptol and icilin [46,47]. Importantly, TRPV1, TRPV4, TRPA1 and TRPM8 are all reachable targets of PM, especially along the respiratory tract in epithelial cells, smooth muscle cells and sensory neurons, and have all been involved in the pathophysiology of the airways, e.g., irritation, inflammation and cough [48,49,50].

**Figure 1:**
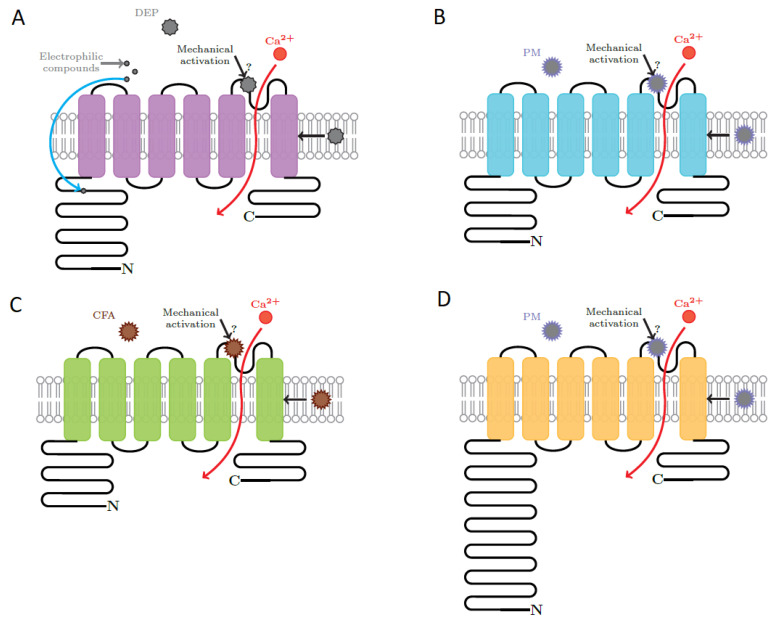
Schematic of the structure of (**A**) TRPA1, (**B**) TRPV1, (**C**) TRPM8 and (**D**) TRPV4 and the representative sites involved in the activation by PM. Modified from [51] (with permission).

The deleterious effects of particulates include inflammation, apoptosis, triggering of asthma, cough, production of mucus, cardiac and respiratory dysfunction, neurodegenerative diseases, reduced cell proliferation, vascular hyperpermeability and edema. Activation of TRP channels by PM is differential and unique as a function of physical and chemical properties of the particles, concentration of particles, structure and function of the channel and numerous interactions either between different particulates or between different channels. Depending on their unique activation mechanism, TRP channels convert the stimulus elicited by NPs into an increase of intracellular Ca^2+^ concentrations, which further activates various pathways. This activation pattern is common to all PM-TRP interactions. The pathways underlying the effect of PM on TRP channels are very complex and understanding them step by step requires an extremely precise control over the experiment. It is because of the complexity of the mechanisms and of the interactions that many of the effects are not yet fully unraveled. This review aims to bring together the results obtained on the topic up to these days and to underline the main effects of different classes of particulates on TRP channels. The structures of TRPA1, TRPV1, TRPM8 and TRPV4 and the proposed sites involved in the interaction with PM are represented in Figure 1 [51,52].

## 2. Effects of PM on TRP Channels

For the purpose of differentiating between the types of particles, PM is further grouped as originating as side-products from industrial applications, functionalized NPs used in medicine and pollutants.

### 2.1. Industrial PM

Particulates resulting from industrial processes are mainly inorganic NPs of silica, titanium or zinc in pure form. They are used in industries such as construction, food, cosmetics, pharmaceutical, pigments, sunscreen and ceramics and are often used as additives to enhance specific properties of products rather than as final products.

#### 2.1.1. Titanium Nanoparticles

Titanium dioxide (TiO_2_) nanoparticles (TiNPs) affect the function of the cardio-respiratory system by interacting with channels of the TRP family at concentrations equivalent to those occupational workers are exposed to. The increase in heart rate induced by medication, the elevation in blood pressure and the immune response triggered by substance P (SP) are higher in rats exposed to TiNPs than in subjects exposed to filtered air, suggesting that these NPs act on nerve fibers. These effects are prevented by prior treatment with ruthenium red, a non-selective TRP channel inhibitor. The action mechanism of TiNPs might involve the production of reactive oxygen species (ROS) and the production of inflammatory factors in the alveoli [53]. TiO_2_ particles can exacerbate asthma symptoms by triggering neurogenic inflammation in lungs. Once inhaled, TiO_2_ particles cause bronchial and lung tissue injury, producing heat, acidosis and various endogenous agonists that are able to activate TRP channels. The influx of Ca^2+^ leads to secretion of inflammatory cytokines and mitochondrial dysfunction, as well as trigger of action potentials that modulate the airway responsiveness. Exposure to TiNPs for 8 h leads to increased Ca^2+^ levels in lung tissue, but the effect cannot be observed in cells exposed for 24 h. Moreover, the expression of TRPV1 and TRPV4 channels is increased in rats exposed to TiNPs for 8 and 24 h [54].

#### 2.1.2. Silica Nanoparticles

TRPM2 has been shown to play a crucial role in the cellular defense mechanism against the toxicity induced by silica nanoparticles (SiNPs). The channel has a protective role and cells with higher levels of TRPM2 show lower ROS and better viability. This is explained by the NAPDH oxidases like NOX2 and NOX4 involved in the production of ROS mediated by TRPM2. Cells with low expressions of the channel show higher NOX2 levels compared to cells expressing more TRPM2, for which the NOX4 levels are decreased. The amount of ROS influences the intracellular Ca^2+^ concentration ([Ca^2+^]_i_) in a directly proportional manner. However, the increase in [Ca^2+^]_i_ is not all due to the activation of the channel, as small percentage of the Ca^2+^ is released from the endoplasmic reticulum. Age also influences the toxicity of the NPs. In mice, younger individuals express lower levels of TRPM2 and, thus, are more susceptible to the negative effects of silica. Overall, SiNPs generate reactive oxygen species that activate TRPM2, leading to Ca^2+^ influx. This, in turn, activates NADPH oxidases, produces more ROS and elevates [Ca^2+^]_i_ even more, resulting in cell death [43].

Another study that addressed the effect of SiNPs on TRPM2 indicates that only 500 nm mesoporous SiNPs have a protective role, but nonporous particles of varying sizes (50–500 nm) do not affect the viability of cells. On the other hand, nonporous particles of different sizes (50–500 nm) show no effect on TRPM2 besides decreased cell viability of cells exposed to 50 nm particles in concentrations higher than 0.75 mg/mL. This result is in contradiction with the previously discussed ones [35].

With regards to TRPM8, another main member of TRPM family, 50 nm nonporous particles have the same effect (increased toxicity), but more pronounced. However, TRPM8 does not play the same protective role that TRPM2 has against 500 nm mesoporous SiNPs, but rather exacerbates their toxic effects. TRPA1 plays a similar role with TRPM2, protecting cells against the toxicity of mesoporous particles, but having no contribution for nonporous silica [35].

Another family of TRP channels interacting with this kind of PM is the TRPV, more specifically TRPV1 and TRPV4. Studies on samples of dessert dust containing silica revealed that Si_2_O activates TRPV1 to a higher extent than TRPM8. TRPV1 is thought to be activated by silica NPs through mechanical perturbations caused by the particles hitting the plasma membrane [51]. When approximately 15 nm NPs of silicon dioxide, titanium dioxide and carbon are compared in terms of their acute response induced (elevation of intracellular levels of Ca^2+^), SiNPs have been shown to be the most potent and TiNPs the least. The increase in intracellular Ca^2+^ has an extracellular origin (through TRPV channels, voltage-gated Ca^2+^ channels VGCC—that might be activated by the membrane depolarization induced by the activation of TRPs or directly by the surface charge of NPs—and leakage through the membrane) and an intracellular one as well (a positive feedback loop of Ca^2+^-induced Ca^2+^ release from the endoplasmic reticulum triggered by the initial influx from the extracellular medium, involving ryanodine receptors) [34,55]. NPs activate TRPV channels by exerting mechanical stretch when they interact with the plasma membrane. Moreover, hypertensive rats (in which the active stretch activated channels are more numerous) show higher Ca^2+^ elevations than normoxic rats [34]. In some cells, an oscillatory pattern can be observed on top of the steady non-oscillatory mobilization from intracellular stores and TRPV4 is responsible for this behavior, but not TRPV1. The oscillations persist after washout and do not depend on NP internalization, but rather on the interaction with the membrane. After a few hours, the cellular homeostatic mechanisms bring the Ca^2+^ concentration to normal levels, regardless of the presence of NPs [55]. TRPV4 shows a not very pronounced protective role in the case of small nonporous particles (50 nm) under certain concentrations. This effect becomes inexistent towards bigger sizes (up to 500 nm). Moreover, the concentrations required to induce a toxic effect increase with particle size. The same effect can be seen in the case of mesoporous particles (500 nm in diameter) [35].

**Figure 2:**
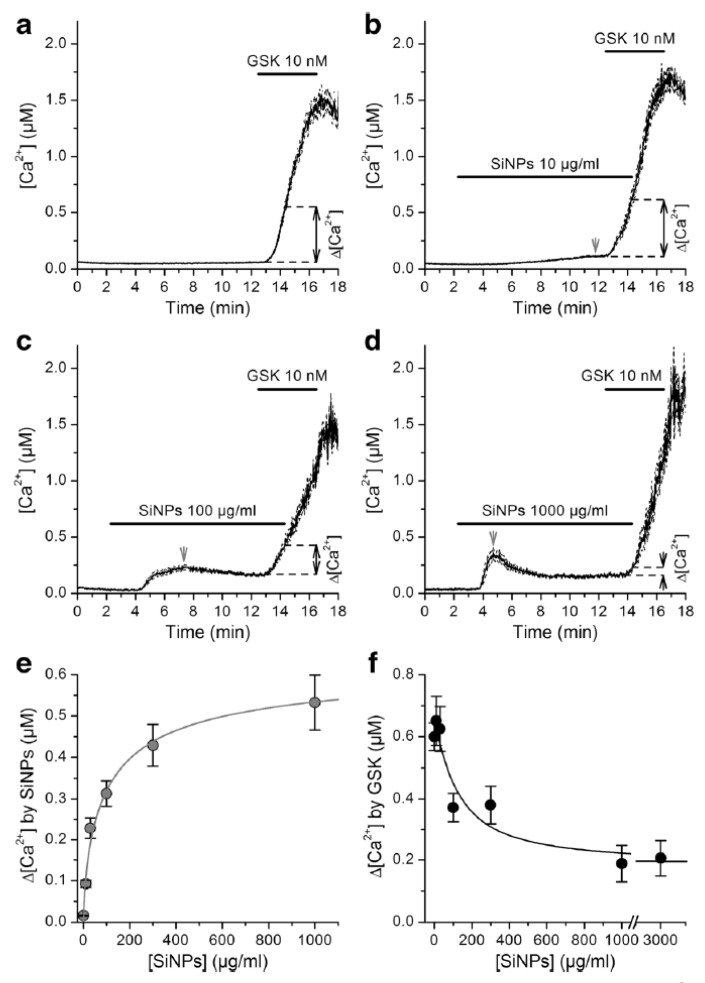
Amorphous silica nanoparticles inhibit the chemical activation of TRPV4. Acute response elicited in cultured human airway epithelial (16HBE) cells by different concentration of 10 nm amorphous silica nanoparticles (SiNPs) and the corresponding TRPV4 inhibition ((**a**): 0 μg/mL, (**b**): 10 μg/mL, (**c**): 100 μg/mL, (**d**): 1000 μg/mL). (**e**) The influx in Ca^2+^ upon SiNPs challenging is concentration-dependent. (**f**) The inhibition of TRPV4 activation by agonist in the presence of SiNPs is concentration-dependent. Reproduced with permission from [24].

Using fluorometric measurements of [Ca^2+^]_i_ it was found that SiNPs inhibit activation of TRPV4 by GSK1016790A in primary cultured mouse tracheobronchial epithelial cells and in cultured human airway epithelial cells 16HBE (Figure 2) [24]. Inhibition of TRPV4 by SiNPs was confirmed in intracellular Ca^2+^ imaging and whole-cell patch-clamp experiments performed in HEK293T cells heterologously expressing this channel. SiNPs enhanced the activation of the capsaicin receptor TRPV1, demonstrating that these particles have a specific inhibitory action on TRPV4 activation. In addition to these effects, SiNPs were found to induce a significant increase in basal [Ca^2+^]_i_, but in a TRPV4-independent manner.

Finally, it was found that SiNPs abrogate the increase in ciliary beat frequency induced by TRPV4 activation in mouse airway epithelial cells. These results showed that SiNPs inhibit TRPV4 activation, and that this effect may impair the positive modulatory action of the stimulation of this channel on the ciliary function in airway epithelial cells. These findings unveiled the cation channel TRPV4 as a primary molecular target of SiNPs.

The mechanism of action of SiNPs on TRPV4 remains unknown. It is unlikely that these NPs reduce channel activation by competitive inhibition because their dimensions are too big compared to the size of the binding site of the chemical agonists site. On the other hand, it may be possible that SiNPs reduce channel activation by GSK1016790A by inducing mechanical stress on the membrane [24].

#### 2.1.3. Zinc Nanoparticles

Zinc is a beneficial metal ion normally found in our bodies, but when levels become too high due to occupational exposure to ZnO or ZnCl_2_, it can cause respiratory inflammation, lung injury and even death. Zinc ions enter the cell via physiological pathways and activate TRPA1 receptors intracellularly, allowing further uptake of Zn^2+^ and Ca^2+^. The disruption of Ca^2+^ homeostasis is associated with cell death and inflammation [56]. In the case of TRPM2, ZnO nitrates a binding site of the channel, leading to the formation of the short isoform, TRPM2-S. This isoform has a weaker interaction with calmodulin (a protein that binds Ca^2+^), causing endoplasmic reticulum stress. In turn, this results in the accumulation of LC3-II, a protein involved in autophagy. The overall effect of the exposure to ZnO is apoptosis by TRPM2-dependent autophagy induction [45].

### 2.2. PM Used in Medicine

Over the time, scientists have learned to take advantage of the penetrating properties of NPs and their deposition and tissue- and molecule-specific interactions for targeted delivery of drugs. This is done by functionalizing the particle surface (or core in the case of porous NPs) with the drugs of interest and other molecules that have the role to enhance the transport and the uptake. Some particles have properties that allow them to be used in the treatment of different diseases or malfunctions.

#### 2.2.1. Bacterial Particles

Bacterial infections cause stiffening of the extracellular matrix and lung inflammation. As TRPV4 is a stretch-activated channel, the presence of bacterial particles activates the channel, inducing Ca^2+^ influx, lipopolysaccharide (LPS)-induced macrophage phagocytosis and inflammatory cytokines. This is true for non-opsonized, as well as for opsonized particles. LPS molecules on the surface of bacterial particles increase activity of TRPV4 in a matrix stiffness-dependent manner [57]. Of note, multiple TRP channels, including TRPV1 [58,59,60], TRPV4 [23,61], TRPA1 [60,62,63,64,65], TRPM3 [60] and TRPM8 [60] have been described as targets of LPS, probably via the induction of modifications in the plasma membrane [66,67]. Therefore, these TRP channels may mediate the action of PM contaminated with this bacterial component.

#### 2.2.2. Carbon Particles

C_60_ fullerenes have been shown to play a role in the treatment of visceral smooth muscle hyperactivity. They penetrate the cell membrane by passive diffusion or endocytosis and have antioxidant, antiviral, antibacterial and antitumor properties. The activation of muscarinic receptors by specific agonists leads to the cleavage of the G-protein, which activates L-type Ca^2+^ channels. These channels are responsible for the depletion of intracellular Ca^2+^ stores and, eventually, the activation of TRPC4 and to a lower extent TRPC6. Fullerenes inhibit this effect almost by half, thus reducing the hyperactivity caused by the activation of the TRPC channels. The inhibition is not direct, meaning that C_60_ fullerenes do not bind to the muscarinic receptor, but they accumulate in the membrane and prevent Ca^2+^ channels to open normally. This, in turn, alters the gating properties of TRPC channels. Moreover, this effect is slow, irreversible and voltage-independent, as fullerenes have been shown not to affect voltage-gated K^+^ channels [68]. Another study showed that charged PC_2_ carboxylate modified particles induce mucin secretion by activating TRPV1 channels. The activation of TRPV1 channels results in Ca^2+^ influx and activates the cAMP/PKA signaling pathway, leading to mucin exocytosis. The production of mucin and the increase in Ca^2+^ and in cAMP are concentration- and time-dependent [69].

#### 2.2.3. Gold Particles

Functionalized gold nanorods (AuNR) and gold nanoparticles (AuNP) have medical applications in cancer treatment. In one study, cationic quaternary amino group based (CTAB) NRs of 70 nm size and anionic NPs of 50 nm diameter have been coated with doxorubicin, neutralizing their apparent surface charge. After treatment of lysosomes, the targets of these nanocompounds, NRs recover their surface charge, while NPs do not completely recover it. The uptake of CTAB AuNRs into lysosomes causes a proton sponge effect. Cations (in this case the NRs) are taken up together with anions (Cl^−^ ions), allowing the pH to remain constant. The excessive uptake of ions causes lysosomal swelling and, eventually, rupture. The result is a release of H^+^ and Cl^−^ to the cytoplasm. Cl^−^ ions activate the TRPM2 channels and the Ca^2+^ channels in the plasma membrane intracellularly, resulting in Ca^2+^ influx and apoptosis. The death of cancer cells is preponderantly (70%) due to the effect of doxorubicin at the cell nucleus and only in a proportion of 30% due to Ca^2+^ influx. The effect is negligible in the case of AuNPs, which do not promote lysosome swelling [70]. Gold nanorods have also been used in a regional anaesthetic (QX-314) hydrogel for their property to produce hyperthermia upon stimulation with near-infrared (NIR) light (photothermal conversion). The release of heat activates TRPV1 channels abundantly found in nociceptors and cause them to open. The hydrogel used has the unique property of solidifying after reaching body temperature and liquefying at temperatures higher than 37 °C. This allows it to be easily injected and only release the anaesthetic in a controlled manner. Once the TRPV1 channels are opened due to the rise in temperature caused by NIR stimulated AuNRs, the anaesthetic can enter the cell and block the voltage-gated Na^+^ channels. The uptake of QX-314 does not take place completely through the opened TRPV1 channels and there is a small temperature-dependent permeation through plasma membrane [32].

#### 2.2.4. Lanthanide Particles

Lanthanide (Ln^3+^) particles such as Nd_2_O_3_, Y_2_O_3_ and upconversion nanocrystals (UCN) can cause inflammation by producing reactive oxygen species generated by NAPDH oxidase inside the cells. Since TRPM2 is sensitive to oxidative stress, the intracellular activation of the channels by ROSs allows Ca^2+^ to enter macrophages and produce inflammation by activating the NLRP3 inflammasome. When coated with RE-1, a small peptide, the formation of ROS and, subsequently, the activation of the inflammasome are inhibited by affecting the endocytosis pathways, but not phagocytosis. Uncoated UCNs are able to elicit a rapid increase in Ca^2+^ levels, followed by a dip and a low-level signal and a steady increase after approximately 100 min. Coating lanthanide particles with this material-specific peptide can reduce the harmful effect of these particulates by 70%, making them more biocompatible [71].

#### 2.2.5. Lipid Particles

Lipid nanocarriers are a very versatile way to transport medication throughout the body. By encapsulating them in lipid vesicles, molecules that would normally react before they reach the target area are protected and their uptake is facilitated by the fusion of the vesicles with the plasma membrane. TRPV1 activity is upregulated in pathological states of neuropathic pain and downregulated in cases of pain caused by injury. In order to reduce pain, the activity or the expression of the channels can be altered. High doses of capsaicin are known to induce TRPV1 defunctionalization and reduce pain, but adverse effect might arise from this. A safer option seems be the slow release of capsaicin from lipid nanocarriers. This prevents the internalization and the degradation of capsaicin [72]. TRPV1 channels can be partially knocked out using short interfering RNA (siRNA), also known as silencing RNA, encapsulated in NPs [73]. Injection with such compounds can efficiently reduce the levels of pain over the course of a few days. TRPM8 activation inhibits the migration of prostate cancer cells. This makes TRPM8 a good candidate for targeted encapsulated drugs. WS12, a TRPM8 agonist, has been shown to preferentially target the fluid regions of plasma membranes, rather than the more rigid ones. When encapsulated in lipid nanocarriers (NCs), the rigid regions shown to be better targeted compared to the application of the non-encapsulated drug. The lipid nanocarriers used in this study were 25 nm in diameter, are negatively charged and are composed by an oily core that contained a mixture of triglycerides with medium chain fatty acids surrounded by a lecithin crown and a hydrophilic surfactant. WS12 NCs act on the extracellular domain of TRPM8 channels and the uptake takes place after 30 min. The advantage of trapping this molecule in NCs is an increase in effectiveness compared to the application of WS12 alone [74].

#### 2.2.6. Iron Particles

NPs containing iron have the unique property of being magnetic, which allows them to react in magnetic and electromagnetic fields by changing the magnetization and/or producing heat. Aligning the magnetic moments of iron oxides (Fe_3_O_4_, Fe_2_O_3_, FeO, MnFe_2_O_4_) causes the NPs to rearrange and to exert mechanical torque forces on the channels (on the condition of being in contact with the plasma membrane). When placed in alternating fields (radiofrequency or near-infrared domain), NPs vibrate and generate heat. The TRPV family is of interest in this context since TRPV1 is activated by heat, and TRPV2 and TRPV4 can be activated by heat and by mechanical forces. The activation of the channels results into a rise in intracellular Ca^2+^ concentration, causing depolarization and triggering of specific cellular processes. This method of remotely controlling the cells allows the release of hormones such as corticosterone, epinephrine and norepinephrine has been achieved up to date [36,75,76,77]. The effect produced depends on the magnetization level (Fe_3_O_4_ has higher magnetization than FeO), the strength of the field and the number of NPs [36,77]. Reaching a high enough concentration of NPs in vivo is a difficult task [76]. Iron oxides can be incorporated with other compounds such as PMAO [36] streptavidin [76], polyethylene [77] and multi-walled carbon nanotubes [75]. It is assumed that the effects of magnetic nanocompounds are rather on the gating of the channel than on the permeability of the lipid bilayer [36].

#### 2.2.7. Poly(amidoamine) Particles

Poly(amidoamine) or PAMAM dendrimers can be used in medicine as nano delivery systems. A study addressing the toxicity of PAMAM dendrimers on different TRP channels reveals that the interactions are selective and depend on the surface functionality of the particles. Two types of PAMAM dendrimers have been used: amine-terminated (positively charged) and carboxyl-terminated (negatively charged). While anionic dendrimers do not produce toxic effects, the cationic ones interact with TRPM2 and TRPA1 channels, but not with TRPM8 and TRPV4. TRPA1 and TRPM2 show reduced susceptibility to the toxicity induced by PAMAM, having a protective role. Moreover, the study suggests that overexpression of these two channels confers resistance to the cells [35].

#### 2.2.8. Poly(lactic-co-glycolic acid) Particles

Poly(lactic-co-glycolic acid) or PLGA are acidic particles that target the lysosomes. TRPML1 channels are expressed in the lysosomal membrane and its activity is reduced by low pH. One of the proteins involved in lysosomal function and autophagy is presenilin1 (PS1). If the function of PS1 is altered, vATPase (responsible for producing the proton gradient) is inhibited and this disrupts the acidification of the lysosome and creates a basic environment, activating TRPML1. The opening of the channel leads to lysosomal Ca^2+^ depletion and, thus, to [Ca^2+^]_i_ increase. This impairs autophagy and leans to neurodegenerative disease [78]. Inhaled PLGA particles containing ruthenium red (RR) can be used to prevent lung edema induced by high pressure mechanical ventilation. TRPV4 is involved in the lung vascular permeation process, which is exacerbated during mechanical ventilation. Inhaled NPs are deposited in the alveoli, where they are phagocytosed by macrophages. RR blocks the mechanically activated TRPV4 channel and prevents ventilator-induced lung injury for 3 days. Although RR blocks the activity of TRPV2 and TRPV4 channels, PLGA particles containing RR only successfully block TRPV4 [37].

#### 2.2.9. Semiconductor Particles

TRPV1 channels are overexpressed in cancer cells. This means that activating this channel can specifically induce cell apoptosis in tumors by Ca^2+^ influx. The rise in [Ca^2+^]_i_ causes mitochondrial membrane depolarization, an influx of Ca^2+^ in mitochondria and, eventually, apoptosis. To activate TRPV1 for this purpose, semiconducting polymers nanoparticles (SPNs) are used to deliver capsaicin to the tumor site. To make sure that capsaicin is not released from SPNs before it reaches the tumor, the complex containing the semiconductive NPs and the agonist is coated in a temperature responsive lipid layer. When irradiated with NIR light in on/off cycles, SPNs release heat, the lipid coating is removed and capsaicin is released. SPNs have been shown to have a better photothermal conversion than carbon nanotubes and gold nanorods [79].

#### 2.2.10. Silica Particles

Silica has biomedical applications in the form of dendritic mesoporous bioactive glass nanospheres. Mesoporous bioactive glass (MBG) particles containing Ca^2+^, silica and doxorubicin (DOX), a drug used in chemotherapy, release Ca^2+^ during the degradation process and extracellular Ca^2+^ levels rise. Normal cells shut down their Ca^2+^ sensitive channels, but in cancer cells TRP channels and Ca^2+^ sensitive receptors are activated. The influx of Ca^2+^ suppresses tumor growth by activating the calpain-1-Bcl-2-caspase-3 apoptotic pathway. Upon the influx of Ca^2+^, calpain-1 is upregulated, Bcl-2 is cleaved and caspase-3 is activated. Compared to mesoporous silica particles, which induce necrosis in both normal and cancer cells, MBGs target tumor cells only. The release of doxorubicin takes place under acidic conditions like in the acidic tumor environment. The efficacy of the NPs is improved by adding the antitumor drug to the MBGs. Two different silica:Ca^2+^ ratios (16:3 in 100 nm NPs and 14:5 in 150 nm NPs) could be assessed by modifying the porosity of the particles, allowing the optimization of treatment. The TRP channels involved in tumor cells exposed to dendritic MBG particles are TRPC6, TRPM4 and TRPM8 [80].

### 2.3. PM Resulting from Pollution

#### 2.3.1. Cigarette Smoke

Cigarette smoke (CS) exposure alters respiratory functions, induces perivascular oedema and emphysema and induces inflammation by elevating the levels of granulocytes, macrophages and lymphocytes. CS contains multiple components such as small particles, free radicals, the unsaturated aldehydes acrolein and crotonaldehyde, the saturated aldehyde acetaldehyde, and nicotine [81,82]. These components can activate TRP channels and cause deleterious effects associated with cancers, heart attack, vascular abnormalities and other pathological processes. TRPA1 is a remarkable airway chemical sensor with roles in both protective reflexes (sneezing, coughing) and pathophysiological responses (inflammation, bronchial hyperreactivity) [40,58]. The components found in CS can activate TRPA1 channels in the plasma membrane of cells located along the respiratory tract. The gaseous phase of CS produces its effects entirely through the activation of TRPA1. Crotonaldehyde is the most potent component, followed by formaldehyde and acrolein. Acetaldehyde has a largely reduced potency compared to the other components. They bind covalently and reversibly to specific cysteine residues in the intracellular N-terminus [51,81,83]. It has been shown that exposure to CS for 14 days elevates the levels of TRPA1 channels in plasma membrane by decreasing IkB kinase (IKK), prolyl hydroxylase domain-containing 2 (PHD2), histone deacetylase 2 (HDAC2) and the nicotinic receptor α7 nAchR and increasing the nuclear translocation of nuclear factor NF-kB and hypoxia-inducible factor HIF1α, which binds to DNA. The increase in TRPA1 mRNA in cells challenged with CS and its decrease after challenge is time- and concentration-dependent. Reactive oxygen species contained in CS elevate the extracellular levels of ROS, activating TRPA1. As a result of increased intracellular Ca^2+^ concentrations, NADPH oxidase is activated, which leads to an increase in intracellular ROS levels, activating the mitogen-activated protein kinase (MAPK) pathway and, eventually, the NF-kB pathway. The activation of NF-kB is also responsible for pro-inflammatory cytokine release, such as interleukin-8 (IL-8) [81,84]. In another study, mice were exposed for 4 months to CS. Emphysema developed at an earlier timepoint in *Trpa1^+/+^* than in *Trpa1^−/−^* animals, but both groups showed significant development by the end of the 4th month. Respiratory deterioration has not been observed in knockout mice. No significant differences were measured between the wildtype and gene-deleted mice when oedema the levels of inflammatory cells were assessed. These two effects peaked by the end of the 3rd month [83]. Acrolein and crotonaldehyde are the components responsible for inflammation induced by the release of substance P-like immunoreactivity (SP-LI) neuropeptide, neurokinin A (NKA) and calcitonin-related gene peptide (CGRP) in neurokinin 1 (NK1)-expressing neurons. The result is bronchi constriction and plasma protein extravasation. TRPV1 is not involved in the release of CGPR, despite the fact that is co-expressed with TRPA1 [58,82]. Unsaturated aldehydes such as acrolein and crotonaldehyde in CS stimulate TRPA1 and the resulting Ca^2+^ influx leads to neuropeptide release. CS can mobilize Ca^2+^ from intracellular stores in an acute concentration-dependent manner. It has been shown that the role of ROS, saturated aldehydes and nicotine in Ca^2+^ mobilization is minor to absent [82]. Cigarette smoke extract (CSE) is responsible for generating ROS and to induce oxidative stress inside the cell and the mitochondria, leading to mitochondrial damage (increased fission proteins and reduced fusion proteins). As a result, TRP channels are activated, intracellular Ca^2+^ levels rise and inflammation occurs, the antioxidant levels decrease and mitochondrial respiratory chain complexes are inhibited [85]. The irritation elicited by acrolein can be attenuated by menthol through the activation of TRPM8 receptor, and not by directly interacting with TRPA1 and TRPV1 [86]. Because TRPA1 and TRPV1 are mostly co-expressed in sensory neurons, stimulation of TRPA1 in capsaicin-sensitized neurons leads to CS-evoked neurogenic inflammation. However, most of the effects produced by CS in cells found in the trachea and larynx co-expressing TRPA1 and TRPV1 result from the contribution of TRPA1, a small part from nicotinic receptors (nAchR) and almost none from TRPV1. Cholinergic receptors are activated by low concentrations of nicotine (in the μM range), while TRPA1 and TRPV1 are activated by concentrations in the μM-mM range. Following the activation of TRPA1 and nAchR, the channels are desensitized and a second exposure to CS does not elicit almost any new response [82,83]. TRPV1 is activated by the ROS produced as a result of CS inhalation, but negligibly by the other CS compounds [87]. The expression of TRPV1 and TRPV4 have been shown to be upregulated in COPD individuals exposed to CS, producing more oxidative stress by Ca^2+^ influx. The oxidative stress is generated by increased levels of nitric oxide and malondialdehyde and decreased levels of superoxide dismutase. The signaling pathway involving IKK, IkB, NFkB and PKB is activated and apoptosis occurs either directly or through inflammatory mediators such as tumor necrosis factor alpha (TNF-α), interleukin 1β (IL-1β), interleukin 6 (IL-6) and phosphor-protein kinase B (p-Akt). These effects can be reversed by flavonoids [79]. The increased expression of TRPV1 and TRPV4 leads to higher intracellular ATP concentrations, P2X-independent activation of pannexin-1 channels and release of extracellular ATP. The result is the activation of P2X channels. Another pathway that leads to P2X activation in the context of exposure to CS involves TRPA1 channels. ROS in CS activate TRPA1 by cyclooxygenase metabolites, resulting in the rise of the [Ca^2+^]_i_. As a consequence, ATP is released and the purinergic receptor P2X is activated, leading to inflammation [81,88,89]. TRPM7 plays an important role in cell proliferation. Exposure to CS results in increased expression and activation of the channel, leading to a higher proliferation rate of airway smooth muscle cells and, eventually, inflammation [90]. TRPM2 channel is not involved in CS-induced inflammatory effects [91]. In contrast with all the previous discussion and evidence of the effect of CS on TRP channels, one study shows that total PM from tobacco preparations does not elicit [Ca^2+^]_i_ increase via TRP channels. Moreover, the study claims that the increase in Ca^2+^ does not have the extracellular or the endoplasmic reticulum as sources, but rather other cytosolic pools [92]. The expression of TRP channels is altered (increased or decreased) in different types of cancerous tissues compared to normal tissues. The changes in channel expression are cancer-specific. As an example, it is known that CS exposure elevates TRPA1 levels and is associated with increased incidence of squamous cell carcinomas. Moreover, endogenous ligands for TRP channels (such as diacyl-glicerol, prostaglandings, protons and other ions) are produced in higher concentrations in cancer cells. CS contains 15 known carcinogenic agents. It does not affect the TRP-encoding genes, but it alters the levels of mRNA and proteins. However, it is not clear if TRP channel expression represents the cause or the effect of cancer development [93].

#### 2.3.2. Coal Fly Ash

Exposure to coal fly ash (CFA) induces pro-apoptotic and pro-inflammatory reactions and leads to pulmonary injury by altering the expression of endoplasmic reticulum stress-associated genes. In addition it triggers the cough reflex, suppressing the respiratory rate and stimulating neurogenic edema [92,94]. CFA particles are mineral-rich particles of 50 nm to 150 μm diameter composed mostly from silicon, aluminum, calcium, iron oxides and carbon. It activates TRPM8, TRPV1 and TRPA1 (in the order of their activation susceptibility), but not TRPM2, TRPV2, TRPV3 and TRPV4 [8,51,94,95,96]. When three of the intracellular amino acids of TRPA1 channel are mutated, CFA has the ability to exert a greater effect on R3C, R58T (N-terminus) and H1018R (C-terminus) variants. These modifications are associated with reduced asthma control in children. The C-terminus mutation also makes the channel inactive for soluble compounds such as acrolein and DTBP. N954T, situated in the transmembrane domain TM6, inactivates the channel for soluble compounds as well, but the response to CFA is not affected. E179K, K186N (in the ankyrin repeats domain), N747A, N753A (in the cell surface N-linked glycosylation site) and N855S (in the cytoplasm between TM4 and TM5) variants all show decreased response to the application of CFA. Moreover, the N855S variant is resistant to soluble agonists. The extent to which CFA activates the channel is only 15% of that by allyl isothiocyanate at the peak activation [96]. CFA is the insoluble component resulting from incomplete fuel combustion. When compared to CFA fractions containing carbon in different proportions (3% vs. 20%) and crystalline silica, Diesel exhaust particles (DEP) activates TRPA1 to a greater extent than the 3% carbon-containing CFA fraction (CFA1), and the 20% carbon-containing CFA (CFA2) and silica do not activate the channel at all. Consistent with other results [96], TRPA1 is mostly activated by soluble electrophilic and non-electrophilic compounds and the contribution of mechanical activation by insoluble electrophilic agonists is lower (approximately 4-fold less activation) [95]. On the other hand, CFA (insoluble electrophilic compounds) is a better activator of TRPV1 than the soluble part of Diesel exhaust (DE) (electrophilic and non-electrophilic compounds) and even crystalline silica (insoluble non-electrophilic). TRPV1 activation by CFA occurs through cell surface interactions with specific amino acid residues in the pore-loop region. The maximal response elicited by CFA takes place at the concentration of 0.73 mg/mL and is mostly due to the CFA1 fraction (3% elemental carbon), followed by CFA2 (20% elemental carbon) and amorphous silica NPs. In contrast, DEP and crystalline silica do not significantly activate the channel. TRPM8 has the greatest affinity for CFA1, followed by TRPV1 and TRPA1 [94].

Studies on the effects of CFA1 on the activity of several TRPV1 variants showed that the Y511A and E649A variants show enhanced activity, C578A, E600V and N604A show decreased activity and that the activation of C621A, A658P and F660A is not altered. The increased activity of the E649A variant can be explained by the absence of electrostatic repulsion between Glu649 and the negatively charged NPs. Elimination of the repulsion increases the reaction to CFA1. In order for the CFA to reach the Glu649 residue, a charge- and mechanosensitive region that includes Cys578, Glu600 and Asn604 has to be displaced by CFA [97]. As for TRPM8 variants, the TRPM8-Δ801 N-terminally truncated variant is responsive to CFA, but at a lesser extent. TRPM8 activation by CFA is augmented by exposure to cold. However, the activation does not require cold temperatures and the potentiation takes place through the PI_2_P binding residue L1008 and is independent of the icilin and menthol binding site (Y745) and the N-terminus region (1–800 amino acids). The R1008Q and L1009R mutations situated in the PI_2_P binding site display reduced responses to CFA by 100% and 60%, respectively. Some of the amino acids in the pore-loop region shown to be involved in TRPM8 activation by CFA are S921, S927 and S932. In order to identify the components of CFA that activate the channel, filtered CFA (97.8% calcium oxides and salts) has been applied to TRPM8. The results show that only calcium oxide particles trigger the opening of the channel [94].

#### 2.3.3. Diesel Exhaust

**Figure 3:**
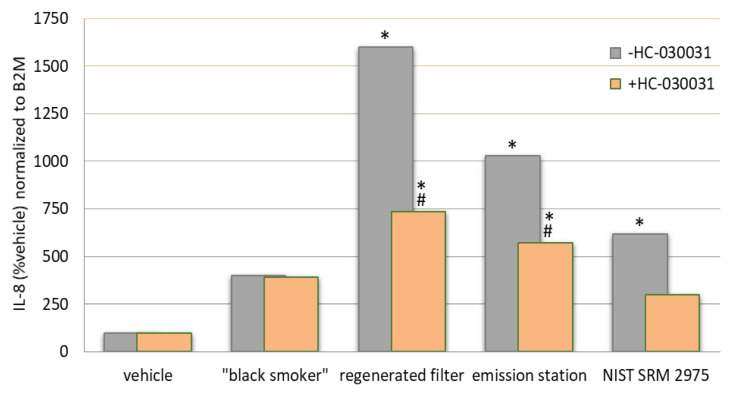
Changes in interleukin 8 (IL-8) mRNA expression as an indicator of inflammation induced by DEP originating from a “black smoker” diesel truck, a diesel exhaust filter regeneration machine, an emission station and NIST SRM 2975. TRPA1 inhibitor HC-030031 reduces the inflammatory effect. The symbols * and # indicate significant induction relative to control and significant inhibition by HC-030031, respectively. Reproduced with permission from [98].

The incomplete combustion of fuels releases fine and ultrafine particles to the environment. DE contains substances such as acetaldehyde, acrolein, hydrocarbons (phenantrene) phosphorus, nickel, cadmium and numerous other substances that make DE a carcinogenic. In nerve cells, DE activates TRPA1, TRPM8 and TRPV1. The Ca^2+^ influx leads to the release of substance P, CGRP and NKA, causing plasma extravasation and edema. In epithelial cells, they activate TRPV1 and TRPV4 indirectly through the protease-activated receptor 2 (PAR2) receptor, leading to chemokine and cytokine release, matrix metalloproteinase-1 (MMP-1) and macrophage activation and lymphocyte chemotaxis. This results in cellular infiltrates and secondary injury. The overall effect of DE inhalation is lung injury [95]. Diesel exhaust strongly activates TRPA1 with 65% of the efficacy of allyl isothiocyanate [96]. The effect of DE varies widely depending on the source, composition and cell type to which it is applied. This translates into unique activation patterns and potencies by different types of DE. Figure 3 suggests that DE in concentrations of 0.38 mg/mL induce TRPA1-dependent inflammation in lung fibroblast cells and that the inflammatory effect can be reduced by TRPA1 inhibitors.

Most of the studies related to the effect of DE on TRP channels focus on TRPA1 because this channel is activated by DEP to a much greater extent than TRPV1, TRPV4 and TRPM8 [51,95]. DEP have been shown to activate TRPA1 at concentrations greater than 77 μg/mL. As mentioned above, TRPA1 activators in DE as mainly soluble electrophilic/oxidizing (acrolein, crotonaldehyde), soluble non-electrophilic (DTBP) and insoluble electrophilic compounds (metals, CFA) [93,96,98]. DEP have been split in 4 fractions based on their polar properties and their potencies of activating TRPA1 have been assessed. The polar and weakly polar fractions have a more pronounced effect than the highly polar and nonpolar fractions and the combined DEP, suggesting that these two components are the ones responsible for DEP-induced TRPA1 responses [99]. Electrophilic agonists bind to C621, C641, C665 and K710 residues on the N-terminus, while non-electrophilic ones bind to the putative menthol/propofol binding site. The activation by mechanical stimuli (insoluble components) is thought to be mediated by ankyrin repeats of the channel. Thus, TRPA1 activation by DEP can be inhibited by mutating the binding sites or by glutathione (antioxidant) [95,98]. A study shows that the R3C and R58T variants of the channel exhibit increased, but not additive response to all three types of agonists. The N855S variant situated between TM4 and TM5, N954T in TM6 and H1018R situated in the intracellular C-terminus seem to be resistant to soluble agonists and show decreased, no response and increased response, respectively to mechanical stimulation by DE. The effect of DEP on the R919Q mutant (in the extracellular pore-loop) is comparable to that on the wild type channel [96].

Polycyclic aromatic hydrocarbons (PAH) can produce cell depolarization indirectly by binding to aryl hydrocarbon receptors, elevating ROS production and activating TRPA1 through covalent modification of cysteine residues, resulting in Ca^2+^ influx. PAH can activate CYP enzymes, which catalyze the production of endogenous lipids and activate TRPA1 and TRPV1, resulting in NF-kB- and NFAT (nuclear factor of activated T-cells)-mediated inflammation [33,100]. Studies on dorsal root ganglion neurons show an increase in [Ca^2+^]_i_, but it has not been established if this effect is fully mediated by the neurons or by non-neuronal cells in the culture [33]. The overall effect of DEP is neurogenic inflammation through release of substance P and neurokinin A (mediated by TRPA1 and TRPV1) and through production of cytokines and chemokines (mediated by TRPV1 and TRPM8), and variations in TRPA1 activation promote differences in lung inflammation and injury [33,95,98]. The activation of TRPA1 by DEP is also responsible for cardiac dysfunction, respiratory responses (by increased release of glutamate triggered by the acrolein component) [95,101], reduced asthma control [96] and locomotor hyperactivity [99]. One major issue of the studies on the effect of DEP on TRP channels is that the concentrations used are higher than those found in high polluted areas and the results might not reflect the actual effects elicited by this form of pollution in humans.

In addition to TRPA1, the organic extract from DE (its carbonaceous core) is also able to specifically activate the proteinase-activated receptor PAR-2, a G-protein coupled receptor that forms part of a signaling complex is located on the cilia of primary human airway epithelia [102]. The detached G_i/o_ component activates PLC and IP_3_ and, subsequently, TRPV4. The Ca^2+^ influx through the channel further activates the mitogen-activated protein kinase (MAPK) signaling, and the phosphorylated extracellular signaling-regulated kinase ERK is translocated to the nucleus, where it activates the respiratory disease-enhancing matrix metalloproteinase MMP-1. This leads to inflammation through enhanced secretion of MMP-1. The chronic obstructive pulmonary disease (COPD)-predisposing variant P19S enhances the activity of MMP-1 by increasing TRPV4 activation.

#### 2.3.4. Ambient Particles

Upon inhalation ambient PM smaller than 2.5 μm (PM2.5) and smaller than 10 μm (PM10) deposit in the alveoli, where they interact with epithelial cells, smooth muscle cells and nerve endings. They are eliminated slowly because the alveoli do not have a clearing function. The composition of ambient particles (AP) varies widely with the region they are collected from and depends on factors such as pollution and natural environment. Nevertheless, they consist of organic carbon, ammonium sulfate, silicon dioxide, and oxides of aluminum, iron, calcium, potassium, magnesium, sodium and titanium [101,103,104]. These compounds can activate TRP channels, being TRPA1 the most susceptible of them, followed by TRPM8, whereas TRPV1 and TRPV4 are activated the least [51].

Asthma is known to be triggered by environmental fine particulates. Studies on asthmatic mice models show that PM exacerbates inflammation by increasing the expression of substance P and greatly increases the expression of TRPA1 and TRPV1 in a time- and concentration-dependent manner. The activation of p-38 MAPK and NF-kB signaling pathways is responsible for the enhanced expression of TRPV1. Neurogenic inflammation can also upregulate TRPV1 expression and function. TRPA1 is thought to initiate the asthma episodes via activation by cigarette smoke, chlorine, aldehydes and scents, while TRPV1 regulates inflammation and cell proliferation. The increased expression of the channels can be reduced by antagonists. Inhibition of TRPA1 alone or TRPA1 and TRPV1 simultaneously shows better effects than the inhibition of TRPV1 alone, suggesting that TRPA1 is more sensitive to the oxidative stress induced by PM2.5 than TRPV1, and that TRPA1 might have a more important role in asthma generation [105,106,107,108,109]. When applied intermittently with acrolein, PM2.5 activates TRPA1 and leads to cardiac desynchrony in mice. The intermittent application elicits unique effects, which differ from the ones produced by individual exposure [101]. Electrocardiograms show that concentrations 30-fold lower than in highly polluted cities elicit an immediate decrease in heart rate, QRS, QT and Tpe intervals and increase in Pdur. These modifications can be successfully prevented by treatment with capsazepine (a non-selective TRPV1 antagonist) after exposure to concentrated ambient particles (CAP2.5). Pretreatment with capsazepine does not reduce PM-induced effects. It is not clear if TRPV1 is involved in these effects or if there are other channels that might be inhibited by capsazepine [106,107]. Exposure to fine and ultrafine PM generates ROS inside the cells. The mechanism by which PM2.5 triggers asthma effects involves the activation of TRP channels by the ROS they produce, the activation of inflammatory pathways as a result of Ca^2+^ influx, the induction of inflammation and the release of neuropeptides that promote airway smooth muscle contraction. The result is cough, dyspnea and hypersensitivity. Intracellular Ca^2+^ signaling mediated by extracellular Ca^2+^ and dependent on phospholipase C (PLC)/IP_3_ pathway can be inhibited by antioxidants, PLC blockers, polyADPR polymerase 1 (PARP) inhibitors and TRPM2 inhibitors (since TRPM2 is activated by oxidative stress). Besides the antioxidant role of melatonin, this molecule has been shown to reduce TRPM2 expression and, eventually, the pro-inflammatory release and chronic cough. PARP is overly produced as a result of the inflammation induced by PM [44,103,105,107,109]. In endothelial cells, PM2.5 concentrations of 10 mg/mL lead to Ca^2+^-dependent protease calpain activation through the activation of TRPM2 elicited by ROS. As a result, the relocation and degradation of tight junction protein Zona occludens-1 (ZO-1) from cell periphery causes reduced ZO-1 levels and endothelial cell barrier disruption. This leads to the formation of gaps and, consequently, to vascular hyperpermeability. Adherens junction proteins do not suffer relocation upon application of PM [104]. Airway vascular permeability is also promoted by the activation of TRPV1 channels [107]. Besides the effects on asthma, ambient PM has negative effects on skin as well. They affect the skin structure, proliferation and differentiation of cells, leading to allergic reactions, aging and delayed wound healing. These effects can be prevented by downregulating TRPV1 expression [105].

#### 2.3.5. Wood Smoke

The combustion of biomasses such as wood results in the spread of PM in the atmosphere that, upon inhalation, it comes in contact with the respiratory tract, exerting negative effects on epithelial and neuronal cells. Wood smoke particles consist of a carbon core covered by polycyclic aromatic hydrocarbons (PAH), aldehydes, ketones, cresol, xylenon, ethylphenon and redox active compounds and have sizes ranging from tenths of micrometer to a few micrometers. They activate TRPA1 through the electrophile/oxidant site (involving C621, C641, C665 and K710 amino acids), through the putative menthol binding site (S873, T874) or by mechanical stimulation. However, the main mechanism of activation is via the electrophile/oxidant actions. It has been shown that WS activates TRPA1 the least, compared to cigarette smoke and diesel exhaust, and that pine smoke is more potent than that of mesquite [110,111]. The activation of the channel is concentration- and size-dependent, with larger particles showing decreased ability to trigger responses. While TRPA1 antagonists inhibit the response to low concentrations of WS, they fail to completely inhibit at the high concentration, suggesting that another WS PM-sensitive Ca^2+^ channel might be involved. The activation is believed to be direct, but indirect activation through oxidative stress bioproducts generated by WS PM is also possible [110]. Wood smoke particles contain humic acid and have the ability to bind cell iron (humic acid has many acidic functional groups and binds cations), resulting in the sequestration of mitochondrial iron from tissues. As a consequence, the expression of divalent metal transporter1 is increased (iron is imported to counterbalance the deficiency), the iron homeostasis is disrupted, ROS are produced in the mitochondria and inflammation occurs, leading to apoptosis [51]. WS particles can activate TRPA1 channels primarily in the plasma membrane, but also in the ER. Moreover, TRPV3 is co-expressed with TRPA1 in the endoplasmic reticulum and its activation is a result of Ca^2+^ influx through TRPA1 channels in the plasma membrane. The result of cytoplasmic Ca^2+^ increase is mucus hypersecretion either directly by the activation of GSK3β and β-catenin signaling pathways or indirectly by loss of cell integrity (involving the shedding of pro-growth factor and the activation of growth factor receptor EGFR expression). By activating TRPA1 in the endoplasmic reticulum, WS PM causes cytotoxicity and endoplasmic reticulum stress by activating the (IRE1)-β, DDIT3, ATF3, HSPA1A, XBP1 pathways. TRPA1 inhibitors attenuate this effect. On the other hand, TRPV3 has a protective role and inhibiting this channel leads to exacerbated endoplasmic reticulum stress and cytotoxicity. Prolonged exposure to WS PM leads to adaptive changes that decrease the deleterious effects of the particles. The transcription is suppressed for TRPA1 and increased for TRPV3. TRPA1 activation stimulates TRPV3, while TRPV3 activation attenuates TRPA1. High intracellular Ca^2+^ levels might promote plasma membrane TRPA1 desensitization. This way, the Ca^2+^-dependent inhibition of TRPA1 by basal TRPV3 activity makes TRPA1 incapable of initiating endoplasmic reticulum stress [112,113]. Separate assessment of molecular compounds shows that cresol does not activate TRPV3, DTBP activates both TRPA1 and TRPV3 and the same is true for xylenon and ethylphenol, but the latter ones have a slightly higher potency and relative selectivity for TRPV3 [111].

## 3. Conclusions

**Figure 4:**
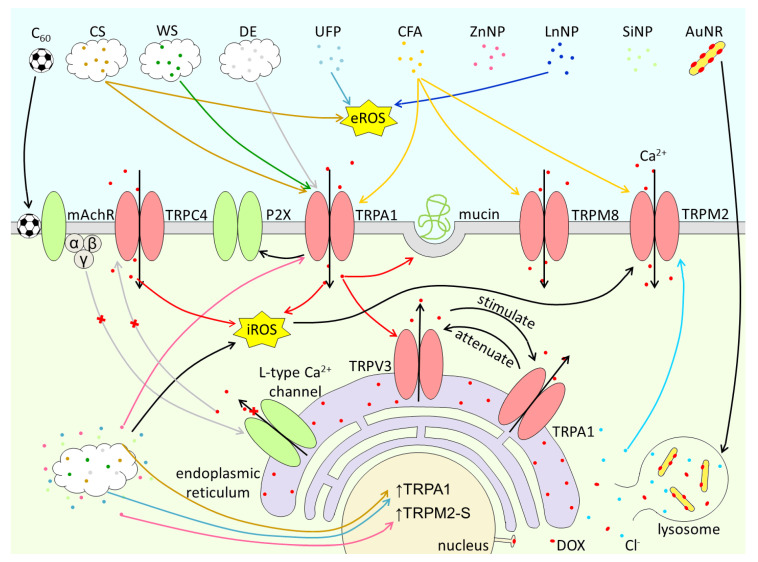
Graphical summary of the activation of TRPA1, TRPC4, TRPM2, TRPM8 and TRPV3 channels by C_60_ fullerenes, cigarette smoke (CS), wood smoke (WS), diesel exhaust (DE), ultrafine ambient particles (UFP), coal fly ash (CFA), zinc NPs (ZnNP), lanthanide NPs (LnNP), silica NPs (SiNP) and DOX-coated gold nanorods (AuNP).

**Figure 5:**
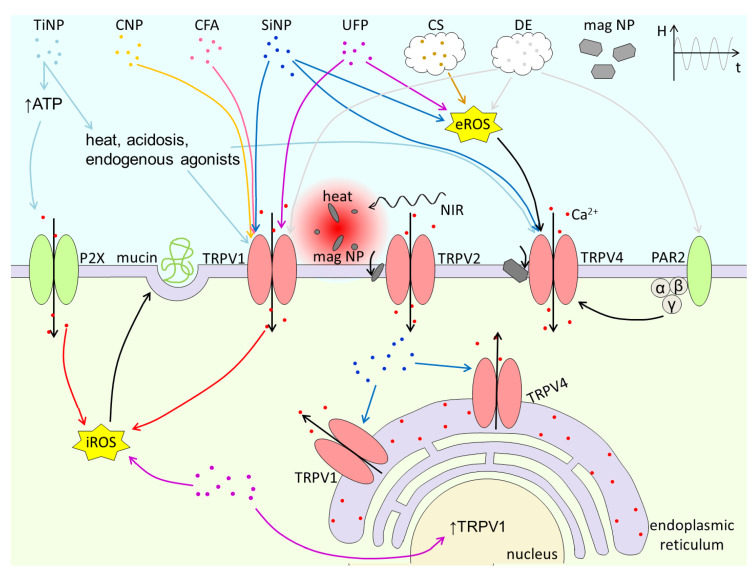
Graphical summary of the activation of TRPV1, TRPV2 and TRPV4 channels by titanium NPs (TiNP), carbon NPs (CNP), coal fly ash (CFA), silica NPs (SiNP), ultrafine ambient particles (UFP), cigarette smoke (CS), diesel exhaust (DE) and magnetic particles (mag NP).

TRP channels encompass at least three properties that make them key targets of PM. First, they are highly expressed in epithelial cells and sensory innervation at the major entry routes into the body (skin, airways, gastrointestinal tract). Second, the have high and broad sensitivity to chemicals contained in PM, and can be activated by mechanical and thermal stimuli. And third, they regulate cellular excitability and intracellular Ca^2+^ concentration and thereby the mechanisms of neurogenic and non-neurogenic inflammation. The know effects of PM on TRP channels are summarized in Table 1, as well as graphically in Figure 4 and Figure 5. The extremely wide variety of chemical composition, size, shapes and textures of PM components increases the possibility that these materials interact with TRP channels. This implies that much work needs to be done to identify and characterize possible PM-TRP channel interactions. Much remains to be done also on the mechanisms underlying these interactions, which according to the available literature can be mediated by: (1) direct activation by chemical components released by PM, (2) activation by ROS induced by the action of PM on other cellular targets, (3) increase in [Ca^2+^]_I_ induced by PM, and (4) detection of perturbations induced in the plasma membrane. Also crucial is to determine the pathophysiological relevance of PM-TRP channels in vivo, especially in the context of human genetic or acquired health conditions that may increase the susceptibility to PM actions [39,52,114,115,116,117,118]. Finally, future studies may reveal exciting possibilities of using engineered nanoparticles for targeted drug delivery to modulate TRP channel function and other biomedical applications.

## Figures and Tables

**Table 1:** Overview of TRP channels targeted by particulate matter.

Type of PM	Target	Effects	Observations
Ambient	General effect	- Directly activated by compounds in AP or indirect activation by produced ROS	
	TRPA1	- Increased expression after prolonged	- Is the most sensitive TRP channel
		exposure	
		- Initiates asthma episodes	
		- Cardiac desynchrony when applied together with acrolein	
	TRPM2	- Activation of the PLC/IP3 pathway	
		- Overproduction of PARP induced by inflammation	
		- Calpain activation	
		- Vascular hyperpermeability by endothelial cell barrier disruption	
	TRPV1	- Increased expression due to activation of p-38 MAPK and NF-kB pathways	
		- Inflammation induced by increased substance P expression	
		- Cell proliferation	
		- Decrease in HR, QRS, QT and Tpe intervals and increase in Pdur (prevented by subsequent capsazepine application)	
		- Airway vascular permeability	
		- On the skin: allergic reactions, aging and delayed wound healing (prevented by downregulation of expression)	
Bacterial	TRPV4	- Increased activity by induced mechanical stress induced by stiffening of extracellular matrix and LPS	
Carbon	TRPC4/TRPC6	- Activation by Ca^2+^ mobilized by muscarinic receptors activation (effect inhibited by C60 fullerenes by accumulation in the membrane and altering gating properties)	
	TRPV1	- Mucin secretion through activation of proton-gated subunit by PC2 particles	
Cigarette smoke	General effect	- Direct activation by compounds in CS or indirect activation by production of ROS	
		- Inflammation through release of substance P and ROS	
	TRPA1	- Activated by the gaseous phase (aldehydes and acrolein) at N-terminal cysteine residues	
		- Prolonged exposure elevates TRPA1 levels by decreasing IkB, PHD2, HDAC2 and α7 nAchR and increasing NF-kB and HIF1α	
		- Bronchoconstriction and plasma protein extravasation as a result of SP-LI, NKA and CGRP release - Respiratory deterioration and emphysema	
		- Ca^2+^ release, not involving ROS, saturated aldehydes or nicotine	
		- Desensitization after first exposure (no second response)	
		- Irritation attenuated by activation of TRPM8	
		- Activation of P2X channels	
	TRPM2		- Not involved in inflammatory effects of CS
	TRPM7	- Altered proliferation induced by inflammation	
	TRPV1	- Apoptosis through IKK, IkB, NFkB and PKB pathways either directly or by TNF-α, IL-1β, IL-6 and p-Akt; effect reversed by flavonoids - Activation of P2X channels	- Almost no contribution to the effects of CS - Mostly activated by ROS in CS - Upregulated expression in COPD subjects
	TRPV4	- Apoptosis through IKK, IkB, NFkB and PKB pathways (effect reversed by flavonoids)	- Upregulated expression in COPD patients
		- Activation of P2X channels	
Coal fly ash	General effect	- Apoptosis and inflammation	
		- Pulmonary injury by inducing endoplasmic reticulum stress	
		- Cough, suppressed respiratory rate and neurogenic edema	
	TRPA1		- The least sensitive to CFA - Activated at the ankyrin repeats domain, glycosilation site and between TM4 and TM5 - Reduced mechanical activation compared to soluble compounds - CFA1 is more potent than CFA2
	TRPM8		- The most sensitive to CFA - Activated by the calcium oxide component
	TRPV1	- Activated by soluble electrophiles, soluble non-electrophiles and insoluble compounds	- Activated at the pore-loop region by insoluble compounds (mechanically) more than soluble ones - CFA1 is more potent than CFA2 - Activation potentiated by cold, independent of putative icilin and menthol binding sites and the N-terminus region
Diesel exhaust	General effect	- Production of ROS	
	TRPA1	- Inflammation through release of substance P and neurokinin A - Cardiac dysfunction, respiratory responses, reduced asthma control and locomotor hyperactivity	- Main channel activated by DE - Mainly activated by soluble polar and weakly polar fractions
	TRPM8	- Inflammation through production of cytokines and chemokines	
	TRPV1	- Inflammation through release of substance P, neurokinin A and though production of cytokines and chemokines	
	TRPV4		- Activated as a result of PAR-2 activation (through PLC and IP_3_)
Gold	TRPM2	- Treatment of cancer through activation by released Cl^−^ from the lysosomes as a result of a proton sponge effect induced by DOX-coated NR	
	TRPV1	- Used in pain control through activation by heat released upon NIR stimulation of NR in anesthetic	
Iron	TRPV1	- Release of hormones through activation by heat dissipated in alternating magnetic field	
	TRPV2		- Activated by heat and mechanical stretch in NIR and magnetic fields
	TRPV4		- Mechanically activated in magnetic field
Lanthanide	TRPM2	- Inflammation induced by ROS production (inhibited by RE-1 coating)	
Lipid	TRPM8	- Inhibited prostate cancer cell migration by encapsulated agonists	
	TRPV1	- Reduced pain by slow release of capsaicin or siRNA encapsulated in vesicles	
PAMAM	TRPA1		- Activated by cationic PAMAM particles - The channel has a protective role
	TRPM2		- Activated by cationic PAMAM particles - The channel has a protective role
	TRPM8		- Not activated by PAMAM
	TRPV4		- Not activated by PAMAM
PLGA	TRPML1	- Impaired development of neurodegenerative diseases by creating an acidic environment in lysosomes	
	TRPV4	- RR-coated particles prevent lung edema induced by mechanical ventilation	
Semiconductor	TRPV1	- Apoptosis by mitochondrial membrane depolarization through irradiation with NIR	
Silica	TRPA1	- Protective role against mesoporous particles, but not against nonporous ones	
	TRPC6	- Cancer treatment through release of Ca^2+^ from Ca^2+^ - and doxorubicin-containing MBG particles	
	TRPM2	- Protective role by influencing NOX-2 and NOX-4 levels, and not through direct effect on Ca^2+^ influx through the channel (effect dependent on porosity and size)	
		- Cell death induced by ROS production	
	TRPM4	- Cancer treatment through release of Ca^2+^ from Ca^2+^ - and doxorubicin-containing MBG particles	
	TRPM8	- Cancer treatment through release of Ca^2+^ from Ca^2+^ - and doxorubicin-containing MBG particles	- Channel overexpression exacerbates the toxic effects of SiNPs
	TRPV1	- Increased Ca^2+^ levels through extracellular influx and release from intracellular stores	- Activated through mechanical perturbations - TRPV1 is more susceptible than TRPM8 - SiNPs are more potent than TiNPs and CNPs - Not responsible for oscillatory behavior
	TRPV4	- Increased Ca^2+^ levels through influx and release from intracellular stores - Responsible for oscillatory behavior (persists after washout, independent on internalization but dependent on interaction with the membrane) - Responsible for inhibited increase in ciliary beat frequency - Normalizing effect on basal intracellular Ca^2+^ concentration	
Titanium	General effect	- Higher BP, substance P release and medication-induced increase in HR (inhibited by prior treatment with RR) - Exacerbated asthma symptoms by induced inflammation - Activation of TRPs by production of ROS, heat, acidosis and various endogenous agonists	
	TRPV1	- Prolonged exposure increases the expression	
	TRPV4	- Prolonged exposure increases the expression	
Wood smoke	General effect	- Activation at the electrophile/oxidant (mainly) and putative menthol sensing sites or by mechanical stress	
	TRPA1	- Apoptosis through iron sequestration by humic acid - Induced cytotoxicity and endoplasmic reticulum stress (activation in endoplasmic reticulum membrane) (effect attenuated by inhibitors) - Prolonged exposure suppresses transcription through desensitization in plasma membrane (attenuation by TRPV3)	- WS is less potent than CS and DE - Direct activation, possibly also indirect through oxidative stress by-products - Activated by DTBP, xylenon and ethylphenon - Activation in plasma membrane and ER Mucus hypersecretion through activation of GSK3β and β-catenin pathways
	TRPV3	- Protective role - Prolonged exposure increases transcription (stimulation by TRPA1)	- Activation in the ER - Activated by DTBP, xylenon and ethylphenon, but not by cresol - Activated by Ca^2+^ influx following TRPA1 activation in plasma membrane
Zinc	General effect	- Respiratory inflammation, lung injury and death	
	TRPA1	- Intracellular activation and further uptake of Zn^2+^ and Ca^2+^	
	TRPM2	- Apoptosis by induced autophagy through the formation of the TRPM2-S isoform	

## Data Availability

Not applicable.
